# Activating Mutations in Protein Tyrosine Phosphatase *Ptpn11* (Shp2) Enhance Reactive Oxygen Species Production That Contributes to Myeloproliferative Disorder

**DOI:** 10.1371/journal.pone.0063152

**Published:** 2013-05-10

**Authors:** Dan Xu, Hong Zheng, Wen-Mei Yu, Cheng-Kui Qu

**Affiliations:** Department of Medicine, Division of Hematology and Oncology, Center for Stem Cell and Regenerative Medicine, Case Comprehensive Cancer Center, Case Western Reserve University, Cleveland, Ohio, United States of America; Emory University, United States of America

## Abstract

Gain of function (GOF) mutations in protein tyrosine phosphatase *Ptpn11* have been identified in childhood leukemias, and these mutations are sufficient to drive the development of myeloproliferative disorder and malignant leukemias in mice. However, the molecular mechanisms by which *Ptpn11* mutations induce these malignancies are not completely understood. Here we report that *Ptpn11* GOF mutations cause cytokine hypersensitivity in hematopoietic cells partly by enhancing the production of reactive oxygen species (ROS). GOF mutations D61G or E76K in *Ptpn11* increased ROS levels in myeloid progenitors but not in hematopoietic stem cells. Increased ROS enhanced cellular responses to cytokines by promoting cytokine signaling. Treatment with an antioxidant partially corrected cytokine hypersensitivity in *Ptpn11* mutant progenitors. Further analyses demonstrated that *Ptpn11* mutations increased mitochondrial aerobic metabolism by interacting with a novel substrate in the mitochondria. This study provides new insights into the pathogenic effects of GOF mutations of *Ptpn11* and implies that antioxidants may have a therapeutic benefit for the leukemic patients with these mutations.

## Introduction

Shp2, a ubiquitously expressed protein tyrosine phosphatase (PTP), is implicated in multiple cell signaling processes [1], [2], [3]. It is normally self-inhibited by hydrogen bonding of the N-terminal SH2 (N-SH2) domain loop to the deep pocket of the PTP domain [4], [5], [6]. Ligands with phospho-tyrosine (pY) residues activate Shp2 by binding the SH2 domains (primarily the N-SH2 domain), thereby disrupting the interaction between N-SH2 and PTP domains and exposing the phosphatase catalytic site [4], [5], [6]. Intriguingly, despite its direct function in protein dephosphorylation, Shp2 plays an overall positive role in transducing signals initiated from receptor and cytosolic kinases [1], [2], [3]. The underlying mechanisms remain elusive. Shp2 interacts with a number of cell signaling intermediates. Of these partners, some are the targets of Shp2 enzymatic activity. However, none of the putative substrates identified to date can fully account for the overall positive signaling effects of Shp2 on the many biological processes with which it has been implicated. It appears that Shp2 functions in growth factor and cytokine signaling in both catalytically-dependent and –independent manners [7], [8], [9].

Shp2 plays a positive role in hematopoietic cell development. *In vitro* erythroid lineage differentiation of embryonic stem (ES) cells with the N-SH2 deletion mutation of Shp2 was severely suppressed and myeloid lineage differentiation was totally blocked [10]. Moreover, the contribution from these mutant ES cells to erythroid, myeloid, or lymphoid cells in the chimeric mice generated from mutant ES cells was undetectable [11], [12]. Recent studies [13], [14] have confirmed that Shp2 is critical for the survival and maintenance of hematopoietic stem cells and immature progenitors. Depletion of Shp2 from adult mice resulted in rapid loss of stem cells and progenitors of all hematopoietic lineages [13], [14]. Importantly, germline and somatic mutations (heterozygous) in *Ptpn11* (encoding Shp2) have been identified in the developmental disorder Noonan syndrome (50%) [15], juvenile myelomonocytic leukemia (JMML) (35%) [16], [17], myelodysplastic syndrome (10%), B cell acute lymphocytic leukemia (7%), acute myeloid leukemia (4%) [18], [19], and sporadic solid tumors [20]. These mutations cause amino acid changes at the interphase formed between N-SH2 and PTP domains, disrupting the inhibitory intramolecular interaction and leading to hyperactivation of Shp2 catalytic activity [16], [21]. In addition, *Ptpn11* disease mutations, especially leukemia/tumor mutations, enhance the binding of mutant Shp2 to signaling partners [22], [23], [24], [25]. Previous studies have demonstrated that these *Ptpn11* gain-of-function (GOF) mutations are sufficient to drive the development of JMML-like myeloproliferative disorder (MPD) and malignant acute leukemias in mice [23], [26], [27], [28], [29]. Nevertheless, as the biochemical basis for the positive role that Shp2 plays in cell signaling and other cellular processes is unclear, the mechanisms underlying the leukemogenesis induced by *Ptpn11* GOF mutations are not well understood. The cytoplasmic function of Shp2 cannot fully explain their pathogenic effects. Emerging evidence has indicated that Shp2 is also distributed to other cell organelles, such as the nucleus [30], [31], [32], [33], [34] and the mitochondria [35], [36]. Understanding of the novel functions of Shp2 in these organelles may shed light on the molecular mechanisms of *Ptpn11*-associated diseases.

## Materials and Methods

### Ethics Statement

All mice used in this study were kept under specific pathogen-free conditions in the Animal Resources Center at Case Western Reserve University. All animal procedures complied with the NIH Guidelines for the Care and Use of Laboratory Animals and were approved by Case Institutional Animal Care and Use Committee.

### Mice

*Ptpn11^D61G/+^* mice [23] were provided by Dr. Benjamin Neel at Beth Israel Deaconess Medical Center. This mouse line was backcrossed with C57BL/6 mice for 4 generations for this study. Backcrossing of *Ptpn11^D61G/+^* with C57BL/6 mice could not be continued because of the complete penetrance of embryonic lethality in F5 *Ptpn11^D61G/+^* mice [37]. *Ptpn11* E76K mutation conditional knock-in (*Ptpn11^E76K neo/+^*) mice generated in our laboratory [29] were backcrossed with C57BL6/J mice for 8 generations for the experiments.

### Flow cytometric analysis and cell sorting

For common myeloid progenitor (CMP), granulocyte macrophage progenitor (GMP), and megakaryocyte erythroid progenitor (MEP) staining and sorting, fresh bone marrow cells were stained FITC-labeled antibodies for lineage markers including Mac-1, Gr-1, Ter119, CD4, CD8a, CD3, and B220 (BD Biosciences, San Jose, CA), c-Kit-APC, Sca-1-PE, CD34-Pacific Blue, CD16/32-PE-Cy7, CD127 (IL-7Rα)-PE-Cy5 (eBioscience, San Diego, CA). Specific cell populations were sorted based on immunophenotypes as previously reported [28]. For LSK cell staining, fresh bone marrow cells were stained with PE-labeled antibodies against lineage markers, Pe-Cy7 labeled anti-Sca-1, APC labeled C-Kit. To measure cellular ROS levels, sorted or stained cells were loaded with 2′-7′-dichlorofluorescein diacetate (DCF-DA) (5 µM) at 37°C for 15 min. ROS (H_2_O_2_) levels in the sorted whole cell populations or in the gated LSK cell population were quantified using flow cytometry.

### Colony-forming unit assay

Freshly harvested bone marrow cells (2×10^4^ cells/ml) were assayed for colony forming units (CFUs) in 0.9% methylcellulose IMDM medium containing 20% fetal bovine serum (FBS), glutamine (10^−4^ M), β-mercaptoethanol (3.3×10^−5^ M), and IL-3 (25 ng/ml). After 10 days of culture at 37°C in a humidified 5% CO_2_ incubator, hematopoietic cell colonies (CFU-GM) were counted under an inverted microscope.

### Generation of bone marrow-derived macrophages

To generate bone marrow-derived macrophages, bone marrow cells were cultured in Dulbecco modified Eagle medium (DMEM) supplemented with 10% FBS and 20% L cell conditioned medium (as a source of mouse colony-stimulating factor 1). After 24 and 48 hours, non-adherent cells were collected and seeded into new tissue culture plates. Following 5 to 7 days of culture, cells were confirmed as macrophages as more than 90% of semi-adherent cells were positive for Mac-1 and F4/80.

### Oxygen consumption and extracellular acidification measurement

Measurement of intact cellular respiration was performed using the Seahorse XF24 analyzer as previously described [38]. Respiration and extracellular acidification were measured under basal conditions, in the presence of mitochondrial inhibitor oligomycin (1 µM), mitochondrial uncoupling compound carbonylcyanide-4-trifluorometh-oxyphenylhydrazone (FCCP) (3 µM), and respiratory chain inhibitor rotenone (1 µM).

## Results

**Figure 1:**
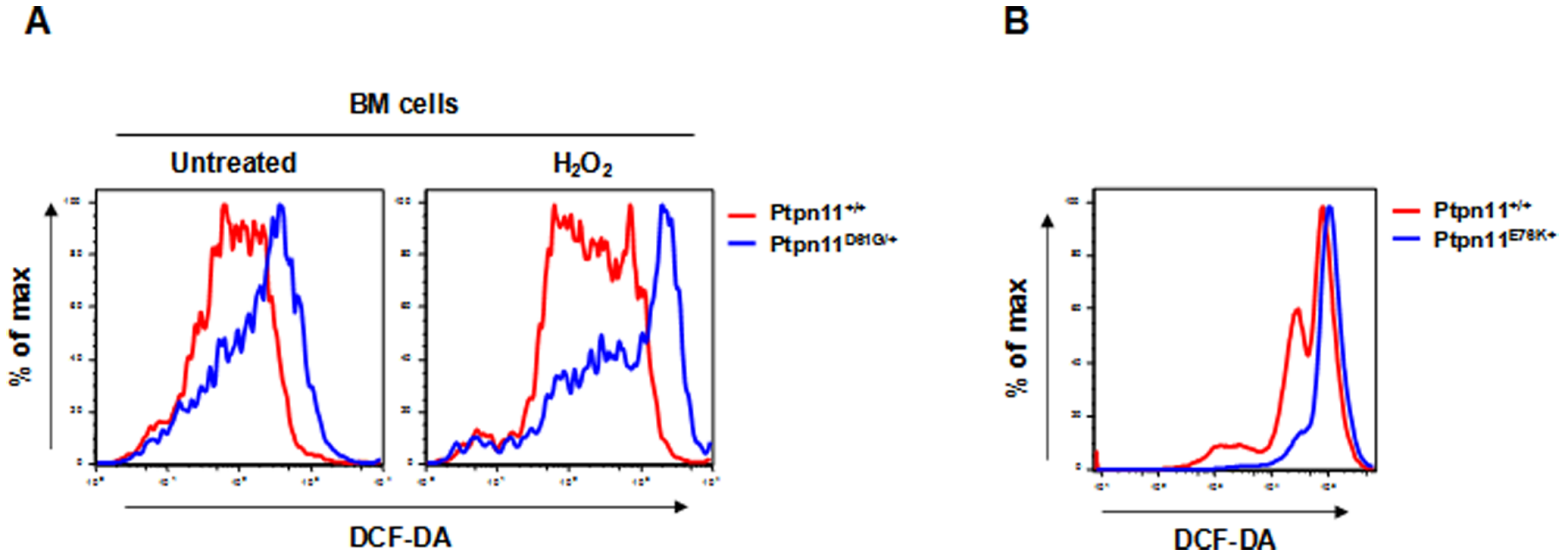
Cellular ROS levels are increased in bone marrow cells of *Ptpn11 GOF* mutant mice. (A) Bone marrow cells freshly harvested from five-month-old *Ptpn11^D61G/+^* and *Ptpn11^+/+^* mice (n = 5/group) were loaded with 2′-7′-dichlorofluorescein diacetate (DCF-DA). In addition, bone marrow cells were treated with H_2_O_2_ (200 µM). Intracellular ROS levels were quantified by FACS. (B) Four-week-old *Ptpn11^E76K neo/+^/Mx1-Cre+* and *Ptpn11^+/+^/Mx1-Cre+* mice were treated by intraperitoneal injection of a total of 5 doses of polyinosine-polycyticyclic acid (pI-pC) (250 µg/mouse) administered every other day over 10 days as we previously described [29]. Twelve weeks after pI-pC treatment, bone marrow cells were harvested from *Ptpn11^E76K/+^* and *Ptpn11^+/+^* mice (n = 5/group). Intracellular ROS levels were quantified by FACS as above.

**Figure 2:**
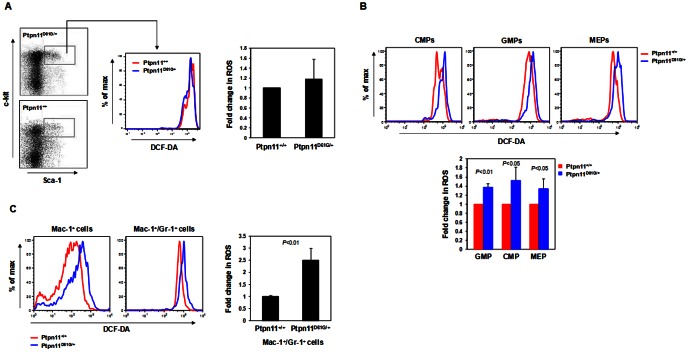
ROS levels are increased in the myeloid cells but not in early stem/progenitor cells of *Ptpn11 GOF* mutant mice. (A) Bone marrow cells freshly harvested from five-month-old *Ptpn11^D61G/+^* and *Ptpn11^+/+^* mice (n = 5/group) were immunostained with cell surface markers. Stained cells were loaded with DCF-DA. Intracellular ROS levels in the gated LSK (Lineage^−^Sca-1^+^c-Kit^+^) cell population were quantified by FACS. (B) Bone marrow cells freshly harvested from five-month-old *Ptpn11^D61G/+^* and *Ptpn11^+/+^* mice (n = 5/group) were immunostained with cell surface markers. CMP (Lineage^−^c-Kit^+^Sca-1^−^CD16/32^med^CD34^+^), GMP (Lineage^−^c-Kit^+^Sca-1^−^CD16/32^high^CD34^+^), and MEP (Lineage^−^c-Kit^+^Sca-1^−^CD16/32^med/low^CD34^−^) cells were sorted by multi parameter FACS. Purified cells were then loaded with DCF-DA. Intracellular ROS levels in these cells were quantified by FACS. (C) Bone marrow cells freshly harvested from five-month-old *Ptpn11^D61G/+^* and *Ptpn11^+/+^* mice (n = 5/group) were immunostained with Mac-1 and Gr-1. Intracellular ROS levels in the indicated cell populations were quantified by multi parameter FACS analyses.

The GOF mutation D61G of *Ptpn11* induces MPD in mice that is characterized by excess expansion of myeloid cells [23]. We found that bone marrow cells isolated from *Ptpn11 D61G* knock-in mice (*Ptpn11^D61G/+^*) displayed markedly increased reactive oxygen species (ROS) levels compared to those in *Ptpn11^+/+^* cells (Figure 1A). Moreover, the capacities of *Ptpn11^D61G/+^* mutant marrow cells to buffer exogenous H_2_O_2_ were reduced. We examined another line of *Ptpn11* GOF mutation (E76K) knock-in mice [29]. *Ptpn11^E76K/+^* bone marrow cells also showed increased ROS levels relative to those in *Ptpn11^+/+^* cells (Figure 1B). To further define the cell populations that were affected by increased ROS, we analyzed ROS levels in Lineage^−^Sca-1^+^c-Kit^+^ (LSK) cells that are enriched with hematopoietic stem cells. As shown in Figure 2A, ROS levels in *Ptpn11^D61G/+^* LSK cells were not changed, nor were ROS levels altered in *Ptpn11^E76K/+^* LSK cells (data not shown). We next examined ROS in myeloid cells at various stages and found that ROS levels in common myeloid progenitors (CMPs), granulocyte macrophage progenitors (GMPs), megakaryocyte erythroid progenitors (MEPs) were all elevated in *Ptpn11^D61G/+^* mice (Figure 2B). Similarly, Mac-1^+^ pan myeloid cells and relatively mature myeloid (Mac-1^+^/Gr-1^+^) cells also showed much higher levels of ROS in *Ptpn11^D61G/+^* mice (Figure 2C). Taken together, these data suggest that GOF mutations in *Ptpn11* increase ROS levels in myeloid progenitors, but not in stem/early progenitor cells.

**Figure 3:**
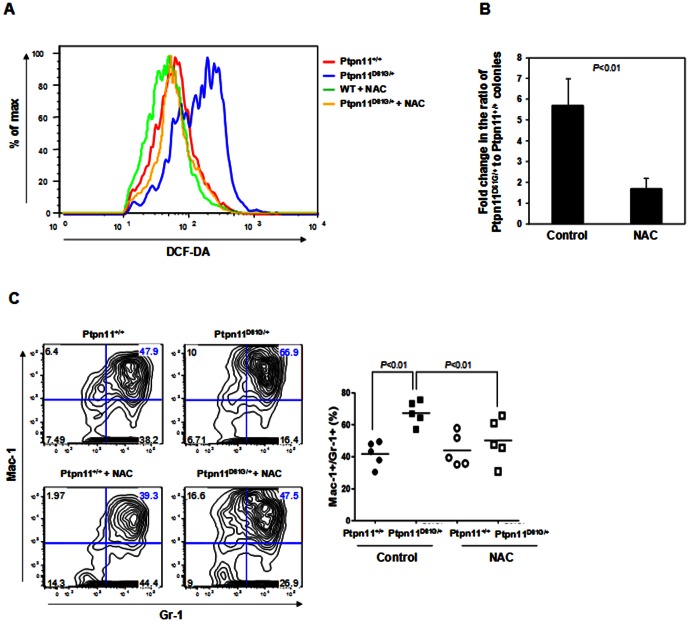
NAC treatment decreases myeloid differentiation of *Ptpn11* GOF mutant cells. (A) Bone marrow cells freshly harvested from five-month-old *Ptpn11^D61G/+^* and *Ptpn11^+/+^* mice (n = 5/group) were cultured in IL-3 (2 ng/ml) containing RPMI 1640 medium with or without N-Acetyl-Cysteine (NAC) (500 µM). After 7 days of culture, intracellular ROS levels were quantified by FACS as described in Figure 1. (B) Bone marrow cells freshly harvested from five-month-old *Ptpn11^D61G/+^* and *Ptpn11^+/+^* mice (n = 5/group) were assayed for colony forming units (CFUs) in 0.9% methylcellulose IMDM medium containing IL-3 (2 ng/ml) with or without NAC (500 µM). Hematopoietic cell colonies (CFU-GM) were counted and normalized. (C) Bone marrow cells freshly harvested from five-month-old *Ptpn11^D61G/+^* and *Ptpn11^+/+^* mice (n = 5/group) were immunostained with cell surface markers. Lineage^−^Sca-1^+^c-Kit^+^ (LSK) cells were sorted by FACS. Purified LSK cells were cultured in IL-3 (0.5 ng/ml) containing RPMI 1640 medium. NAC (500 µM) was added every other day. After 7 days of culture, percentages of myeloid (Mac-1^+^/Gr-1^+^) cells derived from the sorted LSK cells were assessed by FACS analysis (n = 5 per group).

We isolated marrow cells from *Ptpn11^D61G/+^* and *Ptpn11^+/+^* mice and cultured the cells in the presence of IL-3, to which *Ptpn11^D61G/+^* myeloid cells are hypersensitive [23]. After 7 days of culture, *Ptpn11^D61G/+^* cells showed drastically increased total cellular ROS as compared to those in *Ptpn11^+/+^* control cells, consistent with the results shown in Figure 1A. Addition of antioxidant N-Acetyl-Cysteine (NAC) significantly decreased ROS levels in both *Ptpn11^+/+^* and *Ptpn11^D61G/+^* cells. However, ROS levels in *Ptpn11^D61G/+^* cells were still higher than those in control cells (Figure 3A). *Ptpn11* GOF mutation-associated JMML is characterized by cytokine (GM-CSF and IL-3) hypersensitivity in myeloid progenitors [23], [29], [39], [40]. To further determine the effects of the increased cellular ROS levels on cytokine responsiveness, we performed colony-forming unit assays in the presence or absence of NAC. As demonstrated in Figure 3B, following mock treatment, *Ptpn11^D61G/+^* cells were 6-fold more sensitive to IL-3 than *Ptpn11^+/+^* cells. In contrast, a supplement of NAC greatly decreased the sensitivity of *Ptpn11^D61G/+^* progenitors to a nearly normal level. We next isolated LSK cells from *Ptpn11^+/+^* and *Ptpn11^D61G/+^* mice and cultured purified LSK cells in IL-3-containing medium. Myeloid differentiation was examined. As shown in Figure 3C, Mac-1^+^/Gr-1^+^ myeloid cells were substantially increased in mock-treated *Ptpn11^D61G/+^* cells. Seventy percent of the cells derived from mutant LSK cells were Mac-1^+^/Gr-1^+^ myeloid cells whereas myeloid cells accounted for only 40% in the *Ptpn11^+/+^* cell culture. Notably, treatment of NAC largely corrected the aberrantly enhanced myeloid differentiation in *Ptpn11^D61G/+^* LSK cells.

**Figure 4:**
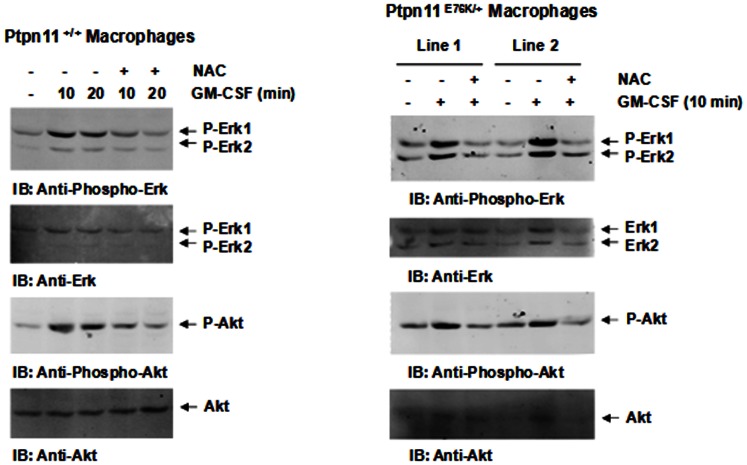
NAC treatment decreases cytokine signaling in *Ptpn11* GOF mutant cells. Four-week-old *Ptpn11^+/+^/Mx1-Cre+* and *Ptpn11^E76K neo/+^/Mx1-Cre+* mice were treated with pI-pC as described in Figure 1B. Twelve weeks after pI-pC treatment, bone marrow-derived macrophages were generated from bone marrow cells isolated from these animals. The macrophages were starved in serum and cytokine-free medium for 5 hours. NAC (1 mM) was added to the culture medium during the last two hours. These cells were then stimulated with GM-CSF (10 ng/ml) for the indicated periods of time. Whole cell lysates were prepared and examined for Erk and Akt activation by immunoblotting with anti-phospho-Erk and anti-phospho-Akt antibodies. Blots were stripped and reprobed with anti-Erk and anti-Akt antibodies to check for protein loading.

To determine the mechanisms by which antioxidant treatment reduced cytokine hypersensitivity in *Ptpn11* GOF mutant cells, cytokine signaling in *Ptpn11^+/+^* and *Ptpn11^E76K/+^* bone marrow-derived macrophages was examined. As demonstrated in Figure 4, treatment of NAC greatly decreased GM-CSF-induced Erk activation in both *Ptpn11^+/+^* and *Ptpn11^E76K/+^* cells. Likewise, Akt activation was also attenuated in NAC treated macrophages. These results suggest that increased ROS in *Ptpn11* GOF mutant myeloid cells contribute to cytokine hypersensitivities by enhancing cytokine signaling.

**Figure 5:**
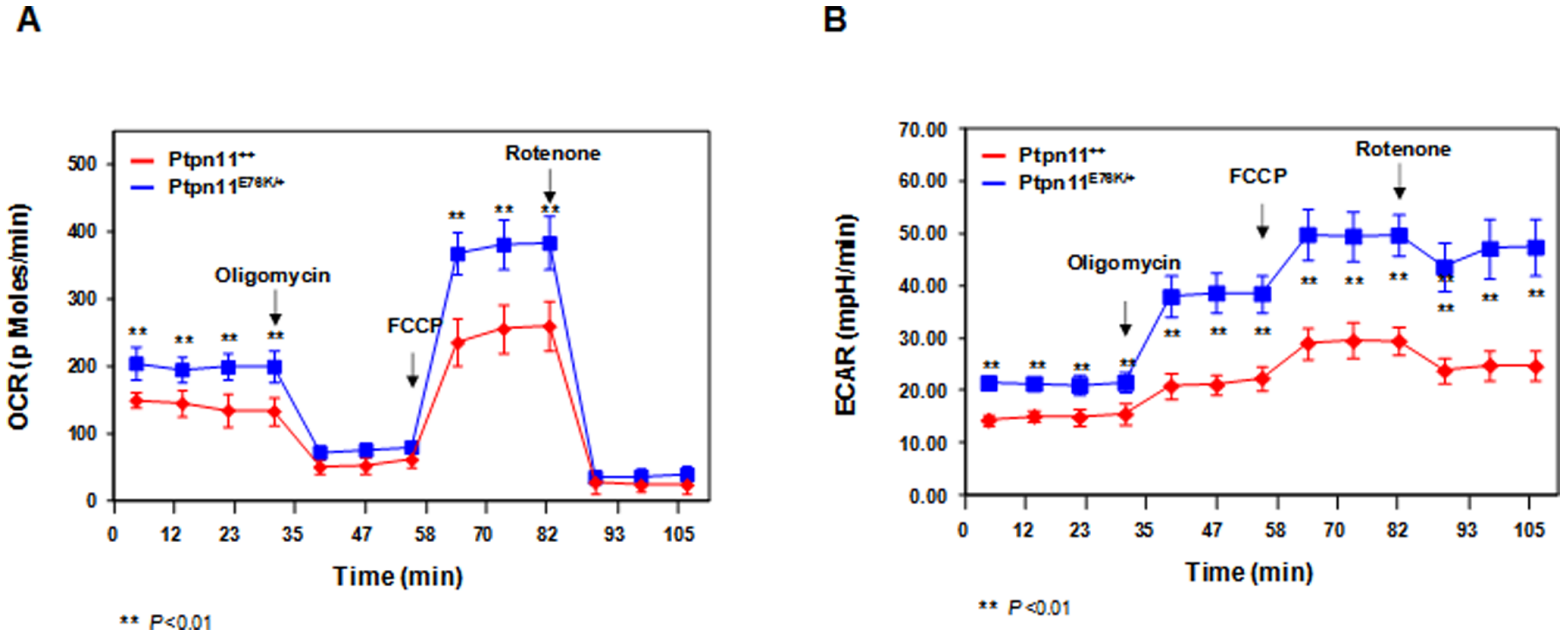
Mitochondrial aerobic metabolism is enhanced by *Ptpn11* GOF mutation. Four-week-old *Ptpn11^E76K neo/+^/Mx1-Cre+* and *Ptpn11^+/+^/Mx1-Cre+* mice were treated with pI-pC as described in Figure 1B. Twelve weeks after pI-pC treatment, bone marrow-derived macrophages were generated from bone marrow cells isolated from these mice (n = 3/group). Oxygen consumption rates (OCR) (A) and extracellular acidification rates (ECAR) (B) of intact macrophages were measured using the Seahorse XF24 analyzer in the presence of the mitochondrial inhibitor (oligomycin, 350 nM), the uncoupling agent (FCCP, 5 µM), and the respiratory chain inhibitor (rotenone, 1 µM).

ROS, such as hydrogen peroxide (H_2_O_2_) and superoxide (O_2_^−^), are produced as a by-product during mitochondrial energy (ATP) production. Shp2 (encoded by *Ptpn11*) is also distributed to the mitochondria [35], [36]. We thus reasoned that Shp2 GOF mutations might increase ROS production by altering mitochondrial metabolism. To test this hypothesis, we assessed mitochondrial function in *Ptpn11* E76K knock-in macrophages by real-time measurement of oxygen consumption in intact live cells. The results showed that *Ptpn11^E76K/+^* cells had increased basal oxygen consumption (Figure 5A). As the addition of the mitochondrial inhibitor oligomycin resulted in a similar and nearly complete reduction in oxygen consumption in *Ptpn11^+/+^* and *Ptpn11* ^E76K/+^ mutant cells, the oxygen consumptions in both cell types under resting conditions appear to be derived almost exclusively from mitochondrial cytochrome chain activity. To measure maximal oxygen consumption, we treated the cells with a mitochondrial uncoupling reagent carbonylcyanide-4-trifluorometh-oxyphenylhydrazone (FCCP). Under the maximally uncoupled conditions, the difference in oxygen consumption between *Ptpn11^+/+^* and *Ptpn11^E76K/+^* cells was even larger (Figure 5A). Subsequent treatment with respiratory chain inhibitor rotenone abolished oxygen consumption to basal levels in both *Ptpn11^+/+^* and *Ptpn11* mutant cells, confirming that the oxygen consumption following FCCP treatment reflects maximal reserve oxygen consumption capacity. Interestingly, measurement of extracellular proton flux revealed that *Ptpn11^E76K/+^* macrophages also had significantly increased extracellular acidification rates (Figure 5B), consistent with these cells also exhibiting enhanced glycolysis.

**Figure 6:**
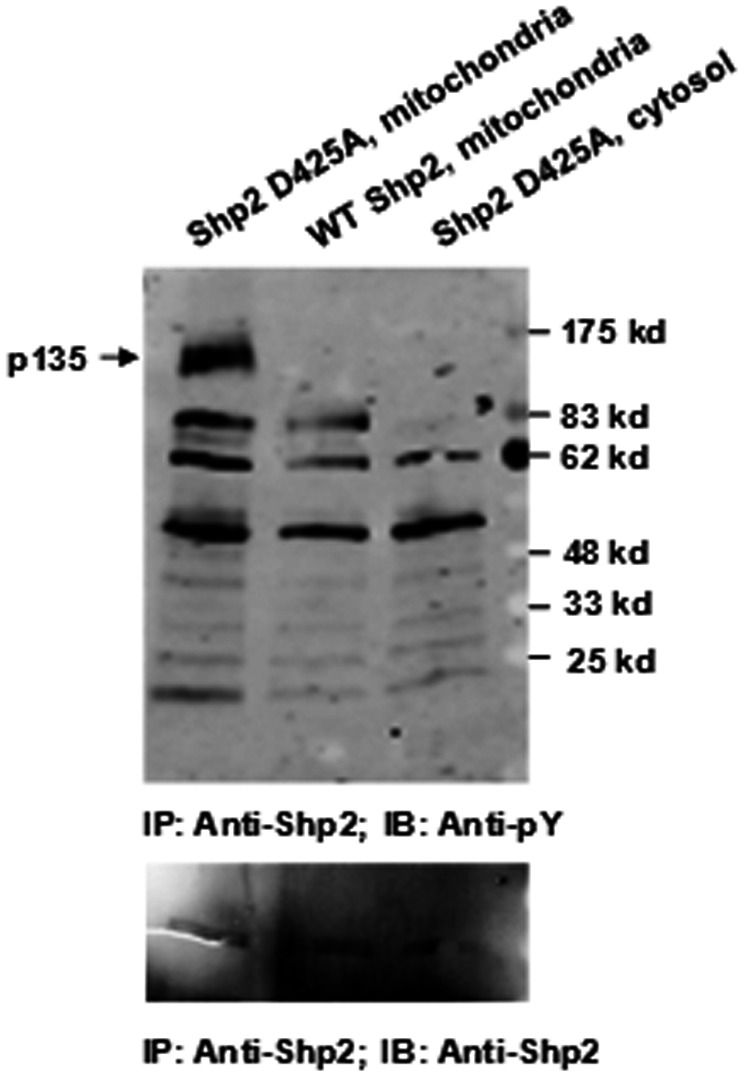
p135 is a potential substrate of Shp2 phosphatase in the mitochondria. BaF3 cells were transfected with Shp2 D425A or wildtype Shp2 expressing plasmids through retroviral mediated gene transfer. Transduced cells were sorted based on green fluorescent protein (GFP) that is expressed under a separate promoter contained in the retroviral vector MSCV. Sorted cell pools were treated with H_2_O_2_ (500 µM) for 20 min. Mitochondria were purified using a kit. Mitochondrial extracts and cytosolic lysates were subjected to anti-Shp2 immunoprecipitation followed by anti-phospho-tyrosine (pY) immunoblotting. Blots were stripped and reprobed with anti-Shp2 antibody.

Shp2 is distributed to the mitochondria in addition to the cytosol and nucleus [35], [36]. Identification of interacting proteins and substrates of Shp2 in the mitochondria would shed light on the mechanisms of the pathogenic effects of *Ptpn11* GOF mutations on mitochondrial function. To achieve this goal, we took advantage of a substrate trapping approach to identify potential Shp2 substrates in the mitochondria. Catalytically-inactive substrate trapping mutant Shp2 D425A [41] or wildtype Shp2 was overexpressed in IL-3-dependent BaF3 cells. These cells were stimulated with H_2_O_2_ and mitochondria were isolated. Anti-Shp2 immunoprecipitation followed by anti-phospho-tyrosine (anti-pY) immunoblotting analysis for the mitochondrial extracts showed that an ∼135 kd tyrosine phosphorylated protein (p135) was trapped by Shp2 D425A, but not wildtype Shp2 (Figure 6). This phosphorylated protein was not present in the Shp2 immunocomplex from the cytosol of the same Shp2 D425A-overexpressing cells. A similar phosphorylated protein was also trapped by Shp2 D425A from the mitochondrial extracts of IL-3-stimulated BaF3 cells (data not shown). Although our mass spectrometric determination of the excised 135 kd band failed to identify this protein in multiple attempts with substantial efforts, p135 is likely one of the Shp2 substrates in the mitochondria and altered functional interaction between Shp2 and p135 may mediate the pathogenic effects of *Ptpn11* GOF mutations on mitochondrial metabolism and thereby the pathogenesis of related malignancies.

## Discussion

In this report, we present the evidence that ROS production in *Ptpn11* GOF mutant myeloid cells was increased and that the elevated levels of ROS contributed to *Ptpn11* GOF mutation-induced excessive myeloid expansion by enhancing cytokine signaling. We also demonstrate that the increased ROS production resulted from increased mitochondrial aerobic metabolism in *Ptpn11* mutant cells. Furthermore, we show that the mitochondrial protein p135 was a potential substrate of Shp2 phosphatase in the mitochondria. These findings provide new insights into the pathogenesis of *Ptpn11* GOF mutation associated hematological malignancies.

GOF mutations in *Ptpn11* appear to contribute to heme malignancies by altering mitochondrial function. Shp2 is mainly distributed in the cytosol, functioning in the cytoplasmic signal transduction. It plays an overall positive role in growth factor and cytokine induced signal transduction although detailed mechanisms are still unclear [1], [2], [3]. GOF mutations in Shp2 increase cell signaling by its elevated catalytic activity as well as enhanced Shp2 binding to pY-containing signaling partners [22], [23], [24], [25]. These mutations induce disease development mainly by altering the cytoplasmic function of Shp2. However, during the disease development and progression, other mechanisms might also be involved. Shp2 is distributed to the mitochondria, specifically the intercristae/intermembrane space [35], [36]. The role of Shp2 in the mitochondria is not understood. The mitochondrial oxidative phosphorylation system provides the vast majority of cellular energy (ATP) and produces ROS. In this report, we show that ROS levels in myeloid cells were markedly increased by *Ptpn11* GOF mutations. ROS in *Ptpn11* mutant stem/early progenitor cells were not significantly changed. This is likely because mitochondria are relatively inactive in stem cells as opposed to those in lineage progenitors as stem cells utilize glycolysis instead of oxidative phosphorylation for energy production [42], [43]. Oxygen consumption of *Ptpn11* GOF mutant myeloid cells showed enhanced aerobic metabolism (Figure 5A). More importantly, the increased ROS production appears to contribute to cytokine hypersensitivity in these cells since treatment with the antioxidant NAC normalized cellular response to IL-3 in colony forming unit assays (Figure 3B) and myeloid differentiation of LSK cells in liquid culture (Figure 3C). Moreover, cytokine signaling was indeed decreased by NAC treatment in *Ptpn11* mutant cells (Figure 4), consistent with previous observations that cellular ROS promote cell signaling [44], [45].

The molecular mechanisms by which GOF mutations of *Ptpn11* enhance mitochondrial aerobic metabolism, however, remain to be further determined. Although Shp2 has been shown to be distributed to the mitochondria [35], [36], little is known about its signaling mechanisms in these organelles. Recently, Lee et al. propose that oxidative phosphorylation complexes might be direct or indirect targets of Shp2 [46]. Using the substrate trapping approach, we now show that the mitochondrial protein p135 is likely one of the substrates of Shp2 in this organelle. Tyrosine phosphorylation of p135 was substantially increased in catalytically-inactive Shp2 D425A mutant-overexpressing cells (Figure 6). It was absent in the Shp2 immunocomplex from the cytosol. This protein does not appear to be the p135 substrate (SHPS-1 also known as SIRPa) of Shp2 previously identified [47] as we were not able to detect this protein using the anti-SHPS-1 antibody in immunoblotting (data not shown). Further efforts in identification of the mitochondrial p135 substrate will shed light on the molecular mechanisms by which Shp2 regulates mitochondrial function and mitochondria-dependent cellular activities, and how GOF mutations of this phosphatase alter mitochondrial metabolism.
